# Will ultra-extended field-of-view scanners be an expensive folly or the next clinical standard for PET/CT?

**DOI:** 10.1186/s40644-022-00486-y

**Published:** 2022-09-06

**Authors:** Rodney J. Hicks, Annick D. Van den Abbeele

**Affiliations:** 1 The University of Melbourne, Melbourne, Australia; 2 Dana-Farber Cancer Institute and Mass General Brigham Hospitals, Harvard Medical School, Boston, MA USA

In a commentary within a landmark supplement to the *Journal of Nuclear Medicine* entitled *Clinical PET: Its Time Has Come,* Henry N. Wagner wrote, “Today’s medical practice is yesterday’s research. The bridge linking the two is technology assessment, which makes possible the acceptance or rejection of new technologies in the practice of medicine” [1]. This statement remains as valid today as it was 30 years ago. In a series of reviews by experts in the field, *Cancer Imaging* will investigate the latest evolution in positron emission tomography (PET) enabled by technological advances in instrumentation that are being adopted by the research community but offer the prospect of a revolution in the way clinical studies are performed.

As our readers will be aware, PET is now embedded in routine oncological practice as a vital diagnostic modality for the evaluation of cancer from diagnosis and staging through treatment planning to therapeutic response assessment and post-treatment surveillance. Combined with computed tomography as a hybrid imaging device, PET/CT has replaced stand-alone PET. Since first introduced in the 1970s [2], ring-configured PET scanners have gone through progressive technological enhancements that have included the removal of septa to allow 3-D imaging [3], improvements in the scintillation detector materials [4], and implementation of time-of-flight capability [5]. All these advances have increased the sensitivity of scanners to detect annihilation photons while improving image quality and quantification [6]. The capability to move patients through the detector ring enabled whole-body imaging [7], which is critical for oncological applications given the potential of cancers to metastasize widely. More recently, replacement of photomultiplier tubes by solid-state digital detectors and adding additional rows of detectors to increase the axial field-of-view (FOV) have further improved the sensitivity of PET devices. The current high-end digital PET/CT systems have performance characteristics unimaginable even a decade ago [8] and industrialization of production has kept the cost of these devices down in real terms, even though they remain significantly more expensive than analog scanners with a restricted axial FOV. A logical evolution of efforts to improve the sensitivity of PET/CT devices is to increase the extent of the body simultaneously covered by detectors.

The first “total-body” PET/CT, the μExplorer (United Imaging, China) was developed by Simon Cherry, Ramsay Badawi and co-workers [9–11]. With an axial FOV of approximately 2 m, this instrument allowed, for the first time, dynamic imaging of the biodistribution of PET tracers throughout the body [12] as well as unparalleled sensitivity for delayed imaging [13]. These features not only provide significant opportunities for radiopharmaceutical development but also for clinical practice by providing very rapid scanning sequences that allow increased patient throughput. Nevertheless, the weight of these devices, their increased dimensions and the cost of manufacture and maintenance as well as the need for an increased number of uptake rooms provide significant challenges for integrating this device into existing PET facilities. An alternative approach has been to develop a PET/CT capable of “total-organ” imaging. With an axial FOV of just over 1 m, the Biograph Vision Quadra PET/CT (Siemens Healthineers) provides similar opportunity to perform dynamic imaging of all the major organ systems from the brain to the pelvis simultaneously [14] and, by acquiring two bed positions or using continuous movement through the scanner, extremely sensitive whole-body imaging. In comparison with a state-of-the-art digital PET/CT the Biograph Vision 600 (Siemens Healthineers), itself a scanner with a substantially higher performance than most of the globally-installed PET/CT base, the ultra-extended FOV of the Quadra scanner yielded an 8–10-fold increment in signal-to-noise ratio (SNR) depending on the radionuclide used [15]. With the same footprint as a standard FOV PET/CT and only moderately higher weight, this scanner can be installed in many existing PET facilities without major modifications but still carries the impediments of requiring more uptake rooms and a higher capital and maintenance cost. A further iteration of this concept has been developed by researchers at the University of Pennsylvania. The PennPET Explorer is a scalable version of an extended FOV PET/CT [16, 17], but it is not yet to our knowledge available commercially.

Given that almost all the evidence validating the utility of PET and PET/CT in the evaluation of a wide range of cancers was obtained using scanners with substantially inferior technical specifications, one might reasonably question whether there is much room to sufficiently improve on the diagnostic performance of PET/CT to justify the incremental complexity and cost of implementing these ultra-extended FOV scanners. If not, they may become a folly installed only in academic institutions which have the financial resources through various granting bodies to acquire and operate these sophisticated devices. However, there are potential reasons why these devices could also offer advantages in routine clinical practice as well as in research. For example, the very high sensitivity of the scanners may allow scanning times to be dramatically reduced, increasing patient comfort and convenience, while also augmenting departmental throughput with sufficient amortization of capital and maintenance costs to make them a viable alternative in sites with high demands for PET services.

With radiopharmaceuticals being a significant consumable cost in operating a PET facility, the increased sensitivity could also be leveraged to reduce administered activities required for high-quality imaging while, at the same time, reducing radiation burden to staff handling an increased throughput of patients, thereby amortizing staffing costs. Where good-manufacturing procedures are required for PET tracers, the ability to affray quality-assurance compliance testing costs across a larger number of patients per production batch provides efficiencies that could further improve cost-effectiveness. Cyclotrons are expensive to install and operate, while some radiopharmaceuticals have such low end-of-synthesis yields as to be uneconomic given the small number of cases that can be scanned from a production. Similarly, radionuclides with a short physical half-life decay while patients are being scanned and, therefore, quicker scans with lower administered activities allows the number of scans that can be performed from a production run to be increased. This is particularly true of carbon-11 tracers, which are attractive options for pharmacokinetic studies, and gallium-68 which are used to radiolabel various agents but most widely for prostate cancer detection with prostate-specific membrane antigen (PSMA) ligand [18]. With germanium-68 generators being in increased demand and having a limited productive yield over time, there are significant potential benefits for clinical accessibility of reducing administered activity and doing quicker scans. These flow-on efficiencies in the radiopharmaceutical supply chain could also have a major impact on the availability of a wider range of PET tracers, particularly those with long physical half-lives that allow regional or international distribution but require relatively low administered activities due to dosimetric considerations. An example of this would be zirconium-89 [19]. Reducing administered activities has additional advantages for imaging children and pregnant women [20], as well as in people susceptible to the development of cancers that could benefit from molecular imaging screening but in whom life-time radiation exposure may be a constraining factor [21].

In a thematic series on total-body PET-CT, we have invited the developers and early adopters of this technology to provide insights into how they are using the unique capabilities of these scanners in research and their routine clinical practice, perspectives on the impact that they will have on radiopharmaceutical development and salutary warnings on the challenges that must be met in processing and analyzing the huge data files generated.

We hope that our readers will look forward to reading articles in this series as much as we are.

*“Every once in a while, a new technology, an old problem, and a big idea turn into an innovation.”*

- Dean Kamen, engineer, businessman and inventor of the Segway vehicle

To our mind, the combination of digital detectors with excellent time-of-flight capability and an extended FOV provide an innovation that addresses the perennial problems of limiting radiation dose to patients and staff members while getting adequate count statistics in a reasonable scan acquisition time. Leveraging economies of scale, greater efficiency in PET services could offset the high-capital cost of this technology. We believe that this will not simply be an evolution of molecular imaging but rather a revolution in the making.
